# Molecular epidemiology of drug-resistant malaria in western Kenya highlands

**DOI:** 10.1186/1471-2334-8-105

**Published:** 2008-07-31

**Authors:** Daibin Zhong, Yaw Afrane, Andrew Githeko, Liwang Cui, David M Menge, Guiyun Yan

**Affiliations:** 1 Program in Public Health, College of Health Sciences, University of California at Irvine, Irvine, CA 92697, USA; 2 Center for Vector Biology and Control Research, Kenya Medical Research Institute, Kisumu, Kenya; 3 Department of Entomology, the Pennsylvania State University, University Park, PA 16802, USA; 4 Center for Infectious Diseases and Microbiology Translational Research, University of Minnesota, Minneapolis, MN 55455, USA

## Abstract

**Background:**

Since the late 1980s a series of malaria epidemics has occurred in western Kenya highlands. Among the possible factors that may contribute to the highland malaria epidemics, parasite resistance to antimalarials has not been well investigated.

**Methods:**

Using parasites from highland and lowland areas of western Kenya, we examined key mutations associated with *Plasmodium falciparum *resistance to sulfadoxine – pyrimethamine and chloroquine, including dihydrofolate reductase (*pfdhfr*) and dihydropteroate synthetase (*pfdhps*), chloroquine resistance transporter gene (*pfcrt*), and multi-drug resistance gene 1 (*pfmdr1*).

**Results:**

We found that >70% of samples harbored 76T *pfcrt *mutations and over 80% of samples harbored quintuple mutations (51I/59R/108N *pfdhfr *and 437G/540E *pfdhps*) in both highland and lowland samples. Further, we did not detect significant difference in the frequencies of these mutations between symptomatic and asymptomatic malaria volunteers, and between highland and lowland samples.

**Conclusion:**

These findings suggest that drug resistance of malaria parasites in the highlands could be contributed by the mutations and their high frequencies as found in the lowland. The results are discussed in terms of the role of drug resistance as a driving force for malaria outbreaks in the highlands.

## Background

Malaria is a major public health problem in sub-Saharan Africa and *Plasmodium falciparum *infection is a leading cause of morbidity and mortality inflicting a huge economic burden in countries where the disease is endemic [1]. It is estimated that death toll of malaria exceeds one million people each year, and the victims are primarily children under the age of five [2]. Until the early 1980s, the African highlands (generally referred to areas of >1,500 m above sea level) were either free of malaria or had very low incidences of the disease; however, since the late 1980s a series of malaria epidemics has occurred [3-9]. Among the many factors that may contribute to the highland malaria epidemics, resistance of the parasites to multiple antimalarials has not been extensively investigated. Resistance to antimalarial drugs is one of the major obstacles for effective malaria control. The first case of chloroquine (CQ) resistance in Kenya was reported in 1977 [10]. In 1993, resistance levels had reached 70% [11]. In 1998, the Ministry of Health of Kenya changed the first line of treatment from chloroquine to sulfadoxine – pyrimethamine (SP; Fansidar^®^) [12]. In 2004, the Ministry of Health of Kenya officially changed the first-line drug to artemether/lumefantrin (Coartem™) [13].

Drug resistance in malaria parasites is associated with genetic mutations in target genes and can be monitored using molecular methods. CQ resistance is determined by the major point mutation at codon 76 of the *P. falciparum *CQ resistance transporter (*pfcrt*) gene [14], which is highly correlated with increased clinical CQ tolerance and treatment failure [14-16]. In addition, point mutations in *P. falciparum *multi-drug resistance gene 1 (*pfmdr1*) (e.g., N86Y, Y183F, S1034C, N1042D, and D1246Y) have been shown to modulate CQ resistance [17] and possibly lumefantrine resistance [18]. Resistance to antifolates is associated with point mutations in the dihydrofolate reductase (*pfdhfr*) and dihydropteroate synthetase (*pfdhps*) genes [19]. Pyrimethamine (PY) resistance is conferred by the key mutation at codon 108 in the *pfdhfr *gene, while additional mutations at positions 51 and 59 increase the levels of resistance [20]. The 164L mutation common in Southeast Asia has been shown to confer PY resistance. Although this mutation is not common in Africa, a recent study by McCollin et al detected the 164L mutation in western Kenya [21]. Similarly, sulfadoxine (SD) resistance is conferred by a key mutation at codon 437 in the *pfdhps *gene and modulated by additional mutations at codons 436, 540, 581, and 613 [22]. Multiple mutations in these two genes result in antifolate treatment failure [23]. Molecular methods have been developed to detect point mutations in genes responsible for drug resistance [24-26], and have been widely used to monitor drug resistance in epidemiological surveys [16]. In this study, we have analyzed the mutations of antimalarial drug resistance genes for CQ (*pfcrt *and *pfmdr1*) and antifolates (*pfdhps *and *pfdhfr*) in samples from patients with acute malaria infections and school children with asymptomatic infections at one lowland and two highland locations in western Kenya.

## Methods

### Sample collection

Sampling in two highland sites (Kisii, elevation ~1600 m above sea level; and Kakamega, elevation 1,480–1,560 m) and one neighboring lowland site (Kombewa, elevation ~1200 m) from June through August 2005 were conducted as a part of malaria surveillance activities. Average annual malaria prevalence among primary school children during the sampling period was 10.3%, 42.7% and 75% in Kisii, Kakamega and Kombewa, respectively. Malaria transmission intensity, measured by entomological inoculation rate (EIR), was 0.4, 16.6 and 31.1 infectious bites per person per year in Kisii, Kakamega and Kombewa, respectively [27]. At each site, 100 samples were collected from patients admitted with acute *P. falciparum* infection at a local hospital and 100 samples from primary school children (age 6–14 years old) with asymptomatic *P. falciparum* infections diagnosed by microscopy. Symptomatic malaria patients were treated by clinicians in the hospital with antimalarial drug, Coartem which achieved cure rates of up to 95%, even in areas of multi-drug resistance. Each sample consisted of ~200 μl of finger-prick blood spotted on filter papers. Filter papers were dried and stored at -20°C until parasite DNA extraction. The human subject protocol involved in this study has been approved by University of California, Irvine (UCI) and Kenya Medical Research Institute (KEMRI). Informed consent was provided by the parents/guardians of the children and assent from the children was obtained prior to the sample collection.

### Parasite DNA extraction and species identification

DNA was extracted from the blood filters using the Saponin/Chelex method [28]. Parasite DNA was extracted from one quarter of a blood spot of about 1 cm in diameter and dissolved in 200 μl of distilled water. Three microliters of the parasite DNA were used as the template for polymerase chain reaction (PCR). To avoid complications from mixed parasite species infections, a nested PCR method was used to verify *P. falciparum *infections and exclude the presence of other *Plasmodium *species in each sample [29]. Approximately 3–5% of samples containing mixed parasite species or other parasite species were identified in all the study sites. Samples containing only *P. falciparum *DNA were used for genotyping analysis.

### Molecular detection of mutations in drug targets

Mutations in the *pfcrt *(K76T) and *pfmdr1 *genes were detected using a PCR-restriction fragment length polymorphism (RFLP) method. The fragment encompassing *pfcrt *codon 76 was amplified and digested with *Apo*I, which cleaves the wild type into 111 and 34 bp fragments [30]. Similarly, fragments containing codons 86, 184, 1034, 1042, and 1246 of the *pfmdr1 *gene were separately amplified by PCR and digested with respective restrictive enzymes as described previously [30-33]. Screening for mutations associated with antifolate resistance at codons 16, 50, 51, 59, 108, and 164 of the pfdhfr gene and codons 436, 437, 540, 581, and 613 of the pfdhps gene was performed by nested PCR and mutation-specific restriction enzyme digestions [34-36].

### Statistical analysis

The difference in frequencies of point mutations in the four aforementioned genes between highland and lowland parasite populations was determined by using the χ^2 ^tests or Fisher's exact test for datasets with sample size less than 5. Yates' correction was applied for the chi-square value, resulting in corrected *P *values. Statistical significance was taken at the *P *= 0.05 level. Association between the different mutations was tested using Fisher's exact test.

## Results

**Figure 1:**
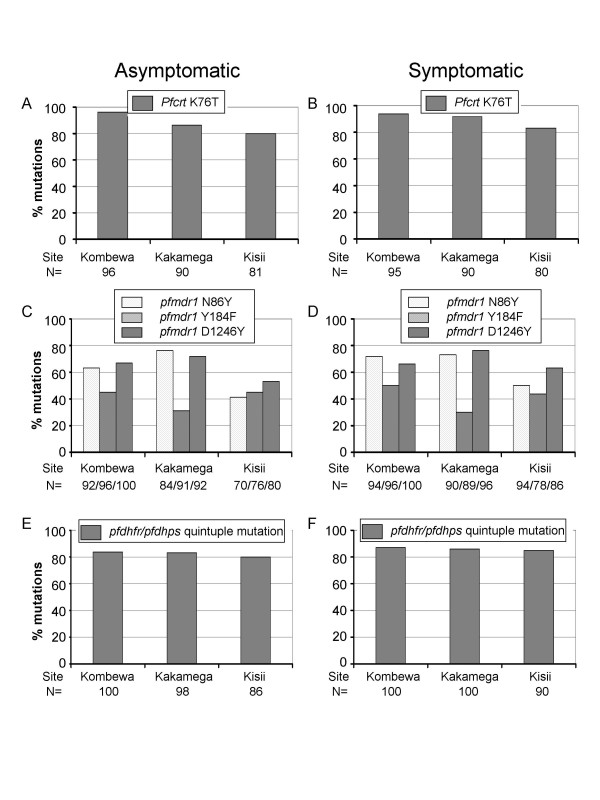
Frequency of mutations in *Plasmodium falciparum *chloroquine resistance transporter gene (*pfcrt*) (A and B), multi-drug resistance gene (*pfmdr1*) (C and D) and quintuple mutations in dihydrofolate reductase (*pfdhfr*)/dihydropteroate synthetase (*pfdhps*) (E and F) genes in symptomatic and asymptomatic volunteers in highland (Kakamega and Kisii) and lowland (Kombewa) sites of western Kenya. The *pfdhfr/pfdhps *quintuple mutation refers to 51I/59R/108N/437G/540E. No mutations were detected at the following codons: *pfdhfr *(A16V, C50R, I164L and S436A), *pfdhps *(A581G and A613S), and *pfmdr1 *(S1034 and N1042D).

A total of 600 *P. falciparum *samples were analyzed for *pfcrt, pfmdr1, pfdhfr*, and *pfdhps *genes. Over 90% of samples were successfully amplified at the 17 test codons, and polymorphisms were detected at 10 of 17 codons screened (Table 1). Consistent with the hyperendemic settings in the study areas, we detected infections by mixed strains at seven of the 17 studied codons, which included codon 76 of *pfcrt*, codons 86, 184 and 1246 of *pfmdr1*, codon 59 of *pfdhfr*, and codons 437 and 540 of *pfdhps *(Table 1). The frequencies of mutant codons at the four genes varied slightly between the study sites, but the differences were not significant (Fig. 1). Mixed-genotype infections at *pfdhfr *codon 59, 437, and 540 of the antifolate target genes were low (<10%) in all samples. Similarly, mixed-genotype infections at codon 76 of the *pfcrt *gene were low (<15%). In contrast, mixed-genotype infections at codons 86, 184 and 1246 of the *pfmdr1 *gene were more frequent (18 – 45%) (Table 1).

**Table 1:** Frequencies of mutations in genes associated with resistance to chloroquine, sulfadoxine-pyrimethamine in *P. falciparum *parasites from symptomatic and asymptomatic volunteers in western Kenya.

Gene	Mutation	Polymorphism	Kombewa		Kakamega		Kisii	
					
			S*	A*	S	A	S	A
*pfcrt*	K76T	Mutant	87	81	86	77	75	70
		Mixed	7	15	6	9	8	10
		Wild-type	6	4	8	14	17	20
*Pfmdr1*	N86Y	Mutant	28	41	28	39	10	23
		Mixed	44	22	45	37	40	18
		Wild-type	28	37	27	24	50	59
	Y184F	Mutant	24	15	7	5	11	26
		Mixed	26	30	23	26	33	19
		Wild-type	50	55	70	69	56	55
	D1246Y	Mutant	24	37	40	28	42	24
		Mixed	42	30	36	44	21	29
		Wild-type	34	33	24	28	37	47
*pfdhfr*	N51I	Mutant	98	96	98	98	96	95
		Mixed	0	0	0	0	0	0
		Wild-type	2	4	2	2	4	5
	C59R	Mutant	81	75	88	79	90	86
		Mixed	4	10	2	7	2	0
		Wild-type	15	15	10	14	8	14
	S108N	Mutant	100	100	100	100	100	100
		Mixed	0	0	0	0	0	0
		Wild-type	0	0	0	0	0	0
*pfdhps*	S436F	Mutant	4	2	4	4	2	2
		Mixed	0	0	0	0	0	0
		Wild-type	96	98	96	96	98	98
	A437G	Mutant	94	95	92	93	88	93
		Mixed	4	5	4	7	2	0
		Wild-type	2	0	4	0	10	7
	K540E	Mutant	88	95	92	91	88	93
		Mixed	10	5	4	9	2	0
		Wild-type	2	0	4	0	10	7

* S – symptomatic; A – asymptomatic.

Note: No mutations were detected at the following codons: *pfdhfr *(A16V, C50R and I164L); *pfdhps *(S436A, A581G and A613S); and *pfmdr1 *(S1034 and N1042D).

### Mutations in CQ resistance genes

Consistent with the past extensive use of CQ in this area, the *pfcrt *76T mutant allele was present in = 80% (including mixed infections with wild type and mutant alleles) of all samples from the three locations (Fig. 1A and 1B). In comparison, mutations at alleles 86Y, 184F and 1246Y of *pfmdr1 *gene were present at lower frequencies, ranging from 30 to 76% (including mixed infections) (Fig. 1C and 1D), whereas no mutation was found at codons 1034 and 1042, which was associated with CQ resistance in South America. The frequencies of mutations were generally not significantly different between the three study sites except *pfcrt *76T and *pfmdr1 *86Y between Kombewa and Kisii (*P *< 0.05), and *pfmdr1 *184F between Kombewa and Kakamega (*P *< 0.01) (Fig. 1E and 1F). Consequently, the genotype 86Y/184Y/1246Y (wildtype at codon 184) was present at a significantly higher frequency in highland than in lowland parasites (*P *< 0.05), whereas the genotype 86Y/184F/1246Y (triple mutations) was significantly more frequent in lowland than in highland parasites (Table 2). Since certain *pfmdr1 *alleles were found associated with CQ resistance, we compared the association between *pfmdr1 *mutations and the major CQ-resistance determinant *pfcrt *76T. When all parasite samples were taken into account, significant associations were found between the *pfcrt *76T and two *pfmdr1 *alleles (184F and 1246Y) (*P *< 0.01). However, *pfcrt *76T was not significantly associated with *pfmdr1 *86Y (*P *= 0.174), the mutation found associated with CQ-resistant field isolates in Asia and Africa.

**Table 2:** Percentage of haplotypes of *P. falciparum *parasites collected from symptomatic and asymptomatic volunteers in western Kenya.

Haplotypes	Kombewa		Kakamega		Kisis	
			
	S*	A*	S	A	S	A
*Pfmdr1*						
86Y/184Y/1246Y	24	26	44	46	38	36
86Y/184F/1246Y	32	34	24	23	16	13
86N/184F/1246D	12	15	0	0	25	27
86Y/184F/1246D	18	10	0	5	1	0
86Y/184Y/1246D	2	0	16	10	1	3
86N/184Y/1246D	6	5	8	10	11	12
86N/184F/1246Y	3	5	4	3	8	5
86N/184Y/1246Y	3	5	4	3	0	4
						
*Pfdhfr/pfdhps*						
Quintuple						
51I/59R/108N/437G/540E	87	84	86	83	85	80
Quadruple						
51I/59C/108N/437G/540E	7	5	3	8	4	4
51N/59R/108N/437G/540E	0	0	4	3	2	6
51I/59R/108N/437G/540K	1	5	1	2	1	2
51I/59R/108N/437A/540E	1	1	0	0	3	4
Triple						
51I/59R/108N/437A/540K	2	3	2	2	5	4
Double						
51I/59C/108N/437A/540K	2	2	4	2	0	0

* S – symptomatic; A – asymptomatic; The mutation amino acids are underlined.

### Mutations in antifolate drug resistance genes

Despite relatively recent deployment of antifolate drugs for controlling malaria in western Kenya, parasites with mutations in the drug target genes, *pfdhfr *and *pfdhps*, were highly prevalent. The mutation at codon 108 (allele 108N) of the *pfdhfr *gene, a major determinant of PY resistance, was ubiquitously present in all samples (Table 1). In addition, mutant alleles at codons 51 and 59 were also detected at high frequencies (85% – 98%). No mutation was detected at codons 16, 50, and 164 of *pfdhfr*. Most samples (90% – 100%) contained the key SD-resistance determinant mutation A437G and K540E in *pfdhps*. In contrast, the mutant 436F allele was rare (< 5%), and mutant 436A allele was not detected in any locations. Further, no mutation was detected at codons 581 and 613 of *pfdhps*.

Increased numbers of mutations at the two antifolate target genes are associated with increased resistance to SD-PY. In the studied samples, single or double mutations were rarely encountered, whereas triple mutations in the *pfdhfr *gene were the most common. When the two genes were combined, quintuple mutations (51I/59R/108N of *pfdhfr *and 437G/540E of *pfdhps*) were found with a high prevalence (80–87%) at the three sites (Fig. 1E and 1F), followed by quadruple mutations (51I/59C/108N/437G/540E, 51N/59R/108N/437G/540E, 51I/59R/108N/437G/540K, 51I/59R/108N/437A/540E) (8% – 16%) (Table 2).

### Mutation frequency difference between asymptomatic and symptomatic samples

The *pfcrt *K76T mutation frequencies were not significantly different in the asymptomatic and symptomatic samples in the lowland Kombewa site (96% vs. 94%; Fisher's exact test, *P *= 0.537) and two highland sites (86% vs. 92% in Kakamega, χ^2 ^= 1.41, df = 1, *P *= 0.235; 80% vs. 83% in Kisii, χ^2 ^0.03, df = 1, *P *= 0.863). Similarly, there was no significantly difference in mutation frequencies of *pdmdr1 *gene at test codons between asymptomatic and symptomatic samples in the lowland (Kombewa: χ^2 ^= 1.44, df = 1, *P *= 0.230 at codon N86Y; χ^2 ^= 0.33, df = 1, *P *= 0.566 at codon Y184F; χ^2 ^= 0, df = 1, *P *= 1.000 at codon D1246Y) and two highland sites (Kakamega: χ^2 ^= 0.07, df = 1, *P *= 0.791 at codon N86Y; χ^2 ^= 0.01, df = 1, *P *= 0.920 at codon Y184F; χ^2 ^= 0.26, df = 1, *P *= 0.610 at codon D1246Y. Kisii: χ^2 ^= 0.87, df = 1, *P *= 0.351 at codon N86Y; χ^2 ^= 0, df = 1, *P *= 1.000 at codon Y184F; χ^2 ^= 1.40, df = 1, *P *= 0.237 at codon D1246Y). Similarly, for the *pfdhfr*/*pfdhps *quintuple mutation frequencies, no significantly difference was found in the asymptomatic and symptomatic samples in both lowland site (Kombewa: χ^2 ^= 0.16, df = 1, *P *= 0.689) and highland sites (Kakamega: χ^2 ^= 0.20, df = 1, *P *= 0.655. Kisii: χ^2 ^= 0.32, df = 1, *P *= 0.572).

## Discussion

In the present study, we analyzed the mutations of four known drug resistance genes in both highland and lowland parasite populations of western Kenya. We showed that the frequencies of key mutations in the *pfcrt*, *pfmdr1*, *pfdhps*, and *pfdhfr *genes that were implicated in resistance to CQ and SP were very high. We further demonstrated that there was no difference in the frequencies of key mutations between symptomatic and asymptomatic malaria volunteers. Our experiment design did not allow us to test clinical or parasitological efficacy in symptomatic infections after treatment; nor was the mutation prevalence/in vivo resistance before 1998 when the national policies of treating uncomplicated malaria were changed to SP was examined. Nevertheless, the big sample size in this study coupled to lack of differences in the frequency of resistant parasite genotypes in the two highland areas and the low land area clearly demonstrates a high frequency of drug resistant mutants circulating in the study areas.

Resistance to CQ is largely determined by the K76T mutation in the *pfcrt *gene, and enhanced by mutations at other sites of this gene and mutations in the *pfmdr1 *gene. Since resistant phenotypes often have fitness costs [37], their prevalence is expected to decline after removal of the selective pressure. For example, the prevalence of mutant alleles of *pfcrt *76T decreased from 64.5% in 2002 to 16% in 2004 and that of mutant *pfmdr1 *86Y alleles decreased from 46.6% to 2.7% two and half year after CQ withdrawal in coastal Tanzania [38]. Kublin et al. reported that the prevalence of the chloroquine-resistant *pfcrt *76T genotype decreased from 85% in 1992 to 13% in 2000. In 2001, chloroquine cleared 100% of 63 asymptomatic *P. falciparum *infections, no isolates were resistant to chloroquine in vitro, and no infections with the chloroquine-resistant *pfcrt *76T genotype were detected [39]. Similarly, Laufer et al. demonstrated that chloroquine was again an efficacious treatment for malaria, 12 years after it was withdrawn from use in Malawi [40]. Although Kenya had a similar drug policy, the *pfcrt *K76T mutation in western Kenya was still predominant. Consistent with the observation of high *pfcrt *K76T mutation frequency in the study sites, *pfmdr1 *mutations that enhance CQ resistance (e.g., 86Y) were also present at relatively high frequencies (37.2 – 45.2%) in western Kenya. This may be partly caused by wide availability and consumption of CQ after it was replaced as first-line antimalarial drug by antifolate drug (SP) in 1998 [12,13]. Our results indicated that parasite populations in western Kenya were still highly resistant to 4-aminoquinoline drugs.

Several years after introduction of SP as the first-line antimalarial drug in Kenya, mutant genotypes have already become common. We found that mutations at codon 108 of the *pfdhfr *gene and codon 437 of the *pfdhps *gene, which are the major determinants for sulfadoxine and pyrimethamine resistance, respectively, were highly prevalent in our study sites. Furthermore, increased resistance to antifolate drugs was correlated with mutations at additional sites, which were also detected in our present study. In Kenya, Nzila et al. [41] reported that the *pfdhfr *triple mutant (codons 108, 51 and 59) was associated with seven-day treatment failure using Fansidar, and this association was strengthened by the presence of double mutations of the *pfdhps *gene at codons 437 and 540. Such "quintuple" mutants were also strongly correlated with Fansidar treatment failure in Malawi [26]. These quintuple mutants have already reached 80–87% prevalence in the parasite populations in western Kenya. This is consistent with the observation in another highland site in Chogoria, near Mount Kenya [42]. Since mutations at different sites of the *pfdhfr *and *pfdhps *genes might be a stepwise process, two mutations at codon 59 of *pfdhfr *and codon 540 of *pfdhps *can accurately predict the presence of this quintuple mutant [26]. The molecular method for monitoring antifolate resistance in *P. falciparum *can thus be simplified to detect the presence of these two mutations. This method has been shown to provide the best means of predicting clinical treatment outcomes in the patient population, which consists primarily of children from endemic areas of Africa [36]. In our study, either of these two mutations has exceeded 75% prevalence in the parasite populations, suggesting that at least 50% of the parasites carry these double mutations. Consistent with other genotype studies of *pfdhfr *from Kenyan isolates, we did not observe mutations at codon 164 [23,41,43], although McCollum et al. identified 164L mutation in several samples in western Kenya [21].

The causes of the malaria epidemics in the East African highlands are complex and could be the result of interactions between environmental changes, vectors and parasites. Antimalarial drug resistance has been invoked as a major factor [9], however the molecular epidemiology of drug resistance in these highlands is unknown. Mbaisi et al. [43] reported a significantly lower prevalence of mutations at codon 437 of the *pfdhps *gene and codons 86 and 1246 of the *pfmdr *gene in lowland (Kisumu) samples than in highland (Kericho, Magadi, and Entosopia) samples in western Kenya, whereas this trend was the opposite for the mutation at codon 436 of the *pfdhps *gene. In contrast, our study did not detect significant differences in frequency of mutations at 14 codons in four drug resistant genes between samples from lowland and highland areas in western Kenya, although the prevalence of mutations at codons 86 and 1246 of the *pfmdr *gene was higher in samples from the lowlands (Kombewa) than in those from the highlands (Kakamega and Kisii). This result suggests either a similar drug selection pressure or significant gene flow between lowland and highland parasite populations aided by human travel [44], as detected by microsatellite and merozoite surface protein 1 and 2 (*msp*-1 and *msp*-2) [45].

## Conclusion

Together with the evidence that there was no significance difference in the frequencies of key resistance-conferring mutations in *pfcrt*, *pfmdr1*, *pfdhps*, and *pfdhfr *genes between symptomatic and asymptomatic malaria infections in western Kenya highland, high frequencies of these mutations in symptomatic and asymptomatic infections suggest that drug resistance of malaria parasites may be an important contributor to malaria-induced morbidity and mortality. However, the role of drug resistance as a driving force for malaria outbreaks in the highlands has not been established. If drug resistance were the main driving force, the lowland and highland sites should expect similar epidemic pattern of malaria incidence. Moreover, drug resistance level should increase over time until a new antimalarial drug is adopted. Thus, the number of malaria patients would be expected to increase gradually over time, but malaria incidence should not exhibit a large inter-annual variation. In contrast, dramatic fluctuations in malaria incidence in the African highlands were observed [9,46,47]. Significant association between climate variability and malaria incidence suggests that climate factors may play an important role in the East African highlands [47]. Further studies are required to examine the interactions between climate factors and drug resistance on malaria incidence in African highlands.

## Competing interests

The authors declare that they have no competing interests.

## Authors' contributions

DZ: Participated in the design of the study, conducted data collection, statistical analysis and drafting of the manuscript. YA: Conducted sample collection and helped with writing the manuscript. AG: Faciliated and conducted field sample collection. LC: Participated in the design of the study, interpretation of data and revising the manuscript. DM: Participated in experimental work and revising of the manuscript. GY: Conceived the study, participated in collection of samples, and participated in manuscript preparation. All authors read and approved the final manuscript.

## Pre-publication history

The pre-publication history for this paper can be accessed here:


